# Mapping the spectrum of psychological and behavioural responses to low‐dose CT lung cancer screening offered within a Lung Health Check

**DOI:** 10.1111/hex.13030

**Published:** 2020-01-21

**Authors:** Sonja Kummer, Jo Waller, Mamta Ruparel, Judith Cass, Samuel M. Janes, Samantha L. Quaife

**Affiliations:** ^1^ Research Department of Behavioural Science and Health University College London London UK; ^2^ School of Cancer & Pharmaceutical Sciences King’s College London London UK; ^3^ Lungs for Living Research Centre UCL Respiratory Division of Medicine University College London London UK

**Keywords:** behavioural sciences, early detection of cancer, lung cancer, mass screening, psychology, smoking

## Abstract

**Background:**

Research on the psychological impact of low‐dose computed tomography (LDCT) lung cancer screening has typically been narrow in scope and restricted to the trial setting.

**Objective:**

To explore the range of psychological and behavioural responses to LDCT screening offered as part of a Lung Heath Check (LHC), including lung cancer risk assessment, spirometry testing, a carbon monoxide reading and smoking cessation advice.

**Methods:**

Semi‐structured interviews were carried out with 28 current and former smokers (aged 60‐75), who had undergone LDCT screening as part of a LHC appointment and mostly received an incidental or indeterminate result (n = 23). Framework analysis was used to map the spectrum of responses participants had across the LHC appointment and screening pathway, to their LDCT results and to surveillance.

**Results:**

Interviewees reported a diverse range of both positive and negative psychological responses, beginning at invitation and spanning the entire LHC appointment (including spirometry) and LDCT screening pathway. Similarly, positive behavioural responses extended beyond smoking cessation to include anticipated implications for other cancer prevention and early detection behaviours, such as symptom presentation. Individual differences in responses appeared to be influenced by smoking status and LDCT result, as well as modifiable factors including perceived risk and health status, social support, competing priorities, fatalism and perceived stigma.

**Conclusions:**

The diverse ways in which participants responded to screening, both psychologically and behaviourally, should direct a broader research agenda to ensure all stages of screening delivery and communication are designed to promote well‐being, motivate positive behaviour change and maximize patient benefit.

## 1 INTRODUCTION

Lung cancer is most frequently diagnosed at an advanced stage (49%‐53% at stage 4)^1^ yet early detection markedly improves prognosis; with five‐year survival increasing from 6% at stage 4 to 82% at stage 1A for non–small‐cell lung cancer.^2^ Implementing low‐dose computed tomography (LDCT) lung cancer screening for high‐risk groups achieves a stage shift to earlier diagnosis.^3^ LDCT screening reduced the relative risk of lung cancer mortality by 20% in the US National Lung Screening Trial (NLST)^3^ and by 26% in the Dutch‐Belgian trial NELSON.^4^ However, there has been concern about the psychological burden of LDCT screening, particularly because the NLST had a high false‐positive rate.^3^ Trials have since differentiated ‘false‐positive’ results (suspicious for cancer) from ‘indeterminate’ pulmonary nodules (usually benign but require surveillance).^5^ Protocols for incidental findings are also evolving, with some reporting only those conditions for which diagnosis would lead to clinical benefit. Evidence from the trial setting suggests any distress induced by these types of results is relatively short‐lived and not clinically significant.^6^, ^7^, ^8^

While research has focused on whether abnormal results cause any clinical psychological morbidity, the ways in which individuals respond to screening may be more diverse and encompass positive as well as negative dimensions. For example, for some people, screening may provoke conscious awareness of risk and distress whereas for others, regular screening may offer a reassuring safety net, providing a positive means of managing risk of lung cancer mortality. The psychosocial component of the Danish Lung Cancer Screening Trial (DLCST) developed a condition‐specific measure of more diverse psychosocial consequences,^9^ which consists of responses spanning social, cognitive and attitudinal domains (eg focus on airway symptoms, existential values). Interestingly, these responses were observed among both ‘screened’ intervention participants and ‘no screen’ control participants,^10^ suggesting aspects of the screening pathway other than the LDCT test itself (eg communicating individual risk status, lung function tests) may have psychological consequences. There are also likely to be individual differences in the way people experience screening and respond to abnormal results, with studies beginning to implicate cognitive risk factors such as higher affective risk perceptions and self‐blame in adverse psychological outcomes.^10^, ^11^

The downstream effects of lung cancer screening on behaviour may be similarly diverse. Ongoing research efforts are directed to understand whether screening undermines or promotes smoking abstinence and how to effectively embed cessation advice and treatment.^12^ However, a qualitative study in the United States found lung screening participants also reported improvements in diet and physical activity,^13^ suggesting the behavioural impact extends beyond smoking. Indeed, evidence drawn from studies of screening for other cancer types,^14^ as well as the diagnostic setting, point to potentially wider‐ranging positive and negative effects across prevention and early detection behaviour. For example, false‐positive breast screening results have been associated with lower subsequent screening uptake,^15^ and all‐clear diagnostic test results as well as negative bowel screening results with reduced concern about symptoms and delayed presentation.^16^, ^17^

The scope of psychological research on the impact of LDCT screening therefore needs expanding to understand the diverse ways in which individuals respond to screening as well as the potential implications for patient experience, well‐being and cancer control. This evidence should inform the design of patient‐centred screening communication and delivery to optimize screening benefit. The present study aimed to explore and map the diverse spectrum of positive and negative psychological and behavioural responses among individuals with indeterminate and incidental LDCT screening results across the entire screening pathway.

## 2 MATERIAL AND METHODS

### 2.1 Participants

Current and former smokers (aged 60‐75) were interviewed four to eight months (Mean: 6 months) after having LDCT screening as part of the Lung Screen Uptake Trial (LSUT).^18^ LSUT was a randomized controlled trial aiming to improve uptake of lung cancer screening by high‐risk individuals (current or recent (quit < 7 years) former smokers, aged 60‐75 years) as part of a ‘real‐world’ demonstration screening service. Individuals were invited to a Lung Health Check (LHC) appointment, which included a medical and smoking history, spirometry and carbon monoxide tests, NCSCT‐accredited ‘Very Brief Advice’ on smoking cessation^19^ and a LDCT scan for those who were eligible based on their risk of lung cancer. Three months after their LDCT scan, purposive sampling was used to recruit a heterogeneous subsample of participants from LSUT who varied in smoking status (current and former), LDCT results (incidental and indeterminate pulmonary nodule) and socio‐economic position (SEP; high and low). A sampling matrix was drawn from a preliminary trial database using these three characteristics. LSUT participants, who fit each of the eight different combinations of characteristics and had consented to further contact, were invited to take part by letter. A researcher (SLQ) checked the demographic and smoking characteristics of those who responded over the phone to ensure each combination of characteristics from the sampling matrix would be represented within the final sample. However, at the time of interview, five of the participants thought to have incidental results described having a clear result and later examination of the final trial database revealed that their results had been downgraded to clear. Although the inclusion of participants with clear results was not part of the planned sampling strategy, data were retained and analysed.

### 2.2 Procedure

Interviews were carried out by SLQ, face‐to‐face (n = 14) or by telephone (n = 14). A semi‐structured topic guide covered (a) experiences of screening and perceived need for support, (b) positive and negative psychological and behavioural responses to the screening process and results, (c) role of perceived stigma and negativity, and (d) anticipated implications for smoking and symptomatic help‐seeking. All interviews were audio‐recorded and transcribed verbatim. Approval was granted by an NHS Research Ethics Committee (15/LO/1186), and participants provided informed consent.

### 2.3 Analysis

Data were analysed in NVivo v.11 by SK using a framework approach^20^ to thematic analysis. The primary objective was to explore the spectrum of positive and negative psychological and behavioural responses to lung cancer screening and their potential influences. SK familiarized herself with the data and developed a coding framework inductively using four transcripts to identify important and recurrent themes. The coding framework was reviewed by SLQ using a subset of transcripts and any disagreements were resolved prior to its application to the remaining data. Following coding, data in each transcript were indexed, charted and summarized systematically into themed matrices so that comparisons could be made by smoking status (current, former) and type of LDCT result (indeterminate, incidental). Six randomly selected transcripts were independently coded by SLQ to review the appropriateness of the indexing codes and themes. Any discrepancies in the coding framework were discussed and resolved.

## 3 RESULTS

### 3.1 Sample characteristics

A total of 129 LSUT participants were invited to take part, 55 responded and 28 were selected for interview. Sample characteristics are presented in Table 1. Participants were current or former smokers who had mostly received either incidental or indeterminate LDCT results. Five participants had received clear screening results.

**Table 1:** Sample characteristics

	**N = 28**
Age, mean (SD)	66 (4)
Gender, n* (%)*	
Female	13 (46)
Male	15 (54)
Ethnicity, n* (%)*	
White	24 (86)
Non‐white	4 (14)
Education, n* (%)*	
No formal qualifications/ Left school at or below age 15	14 (50)
Completed GCSE/O Levels/A Levels or equivalent/ Further	6 (21)
Completed University degree	8 (29)
Marital status, n* (%)*	
Married/co‐habiting	10 (36)
Single/Separated/Divorced/Widowed	18 (64)
Smoking status, n* (%)*	
Current smoker	18 (64)
Former smoker	10 (36)
Pack years^a^, mean (SD)	48 (35)
Index of Multiple Deprivation (IMD) rank quintile, n* (%)*	
Quintile 1 (most deprived nationally)	14 (50)
Quintile 2 (second most deprived)	14 (50)
LDCT result, n* (%)*	
Indeterminate	10 (36)
Incidental	13 (46)
Clear	5 (18)

Abbreviation: LDCT, low‐dose computed tomography.

a One pack‐year is equivalent to smoking 20 cigarettes of cigarettes every day for one year.

### 3.2 Overview of themes

The presentation of results is structured by three main themes: (a) psychological responses, (b) behavioural responses and (c) factors influencing psychological and behavioural responses. Psychological responses varied very clearly over the course of the screening pathway, so subthemes relate to the stages of the screening process from LHC invitation to receipt of results. Behavioural responses covered a number of behaviours as well as anticipated behaviours that participants discussed in relation to their experience of screening. In the third theme, we have drawn out factors (independent of stage on the pathway) that appeared to explain the range of positive and negative psychological and behavioural responses observed. The three main themes and subthemes are illustrated in Table 2 and further described below with short illustrative quotes. Reference codes represent participant number (P), type of LDCT result (Nod/Inc/Cle = Indeterminate pulmonary nodule/ Incidental finding/ Clear result) and smoking status (CS/FS = current/former). Our coding frame is also available in File S1.

**Table 2:** Thematic structure of the data

Psychological responses
Prior to Lung Health Check (LHC) When told about LDCT eligibility Spirometry test results Waiting for LDCT results Receiving LDCT results Waiting for and undergoing follow‐up tests
Behavioural responses
Future anticipated screening participation Future anticipated symptomatic help‐seeking Smoking behaviour Other health behaviours
Factors influencing psychological and behavioural responses
Existing concerns about lung health and smoking history Social support Stigma and self‐blame Negativity and fatalism Competing priorities

### 3.3 Psychological responses

Psychological responses varied along the screening pathway and are presented for each stage.

#### 3.3.1 Prior to the Lung Health Check (LHC)

Most regarded their invitation to the LHC as a positive occurrence and were ‘glad it was happening’ [P16/Cle/CS], as well as feeling cared for and finding the offer ‘comforting*’* [P26/Inc/CS]. Regardless of smoking status, a minority described how they ‘didn't really feel concerned’ [P08/Nod/FS] or ‘worried’, with some reporting feeling *‘*fine’*, ‘*relaxed’ and *‘*happy about going’. Some looked forward to the appointment because they were ‘interested to know what the results would be’ [P04/Inc/CS]. Indeed, the LHC appeared to increase some participants' perceived control over their respiratory health by providing an opportunity to address pre‐existing concerns (‘to find out if I've got anything wrong with my lungs’ [P22/Nod/FS]), or to establish the cause of medically undiagnosed symptoms (‘find what's causing this pain’ [P17/Cle/CS]). In this way, screening seemed worry‐reducing by setting a process in motion for those who were *‘*more anxious before the letter arrived’ [P01/Nod/CS]. One participant described how the invitation to LDCT screening was *‘*empowering… [the LHC offer was saying] you are important and we want you not to suffer… bringing you out of this enclosed pen’ [P19/Inc/CS].

There was some evidence that the invitation letter provoked concern about lung health and risk of lung cancer in the lead up to the appointment, including greater attention to previously unacknowledged symptoms: ‘I would be more quick to notice if I was out of breath’ [P18/Inc/CS]. A few current smokers reported feeling ‘a bit shocked’ [P28/Inc/CS] or ‘a bit anxious’ [P16/Inc/CS) when they received the invitation. These feelings appeared to stem from the expectation of ‘bad news… Here's where they find out I've got [cancer]’ [P16/Inc/CS]. One noted their anxiety was ‘because of smoking’ [P23/Nod/CS].

#### 3.3.2 When told about LDCT eligibility

Some participants expected to be offered a LDCT scan at their LHC, because this possibility was outlined in the invitation materials or because they perceived their lung health as poor. Others had not expected to be offered a scan, which came as ‘a bit of surprise’ [P13/Nod/FS]. Apprehension about being scanned was only raised by one participant who was concerned about the potential result. Instead, participants described reacting positively to being eligible for a LDCT scan; seeing it as an opportunity to find out whether something was wrong with their lungs.

#### 3.3.3 Spirometry test results

The type of spirometry result appeared to have the potential to positively or negatively affect participants' perceptions of their risk of lung cancer and expectations of the type of LDCT result they would receive. Participants described feeling ‘more positive’ or ‘happy’ following a normal spirometry result and subsequently felt optimistic about their LDCT result. An abnormal spirometry result caused disappointment, with participants stating how they had felt ‘terrible’ or how ‘this is when [they first] got worried’ [P02/Inc/FS]. There was evidence that the effect of spirometry results continued in the longer term, affecting how participants responded psychologically to their LDCT result and any follow‐up tests. One participant described how the abnormal spirometry exacerbated their concern about their indeterminate LDCT result, ‘playing on my mind constantly with now waiting for the [follow‐up] test’ [P13/Nod/FS].

#### 3.3.4 Waiting for LDCT results

Extreme anxiety concerning the possible LDCT result was rare. When anxiety was reported, this appeared to be due to fear of ‘what the results would show’ [P03/Inc/CS] and some worry was considered normal. Instead, some participants expressed how they had felt ‘neutral’ or ‘fine’ while waiting and thought about the potential outcome only occasionally, with some deciding: ‘it's not really worth worrying until you actually know’ [P24/Cle/FS].

#### 3.3.5 Receiving LDCT results

Some perceived that their risk of lung cancer remained just as high following their LDCT results as it had been before: ‘I still think it's pretty high’ [P15/Nod/FS]. However, despite knowing that ‘lesions aren't necessarily cancerous’, there was evidence that some participants with an indeterminate result became preoccupied with the thought that it ‘might be cancerous… that little dot on my lung’ [P06/Nod/CS]. Those not expecting an abnormal result appeared to be more adversely affected, perhaps because they were less psychologically prepared: ‘I wasn't really expecting something like that’ [P13/Nod/FS]. There were some however, who perceived their risk as lower, but primarily due to giving up smoking, the LDCT scan diagnosing a previous health concern or because they now paid greater attention to their lung health.

Incidental findings by contrast were frequently interpreted positively as an ‘all‐clear’ for cancer. There were descriptions of *‘*having dodged a bullet’ [P19/Inc/CS], feeling ‘over the moon’ [P11/Inc/CS] or being given a ‘second chance’ [P25/Inc/CS]. Indeed, some participants were ‘relieved there was no cancer’ [P04/Inc/CS] and reassured: ‘like getting an MOT to say, well you're cool for the next couple of years… for the rest of your life you're not at a disadvantage’ [P19/Inc/CS]. For most, this reassurance appeared to be appropriate and acknowledged as temporary: ‘being a smoker you're looking at a matter of time’ [P16/Inc/CS]. One participant seemed over‐reassured, describing how they ‘could afford to be reckless a little bit longer’ [P04/Inc/CS]. Nevertheless, there was also evidence that incidental findings drew attention to other ways in which smoking causes risk to health beyond lung disease. For one participant diagnosed with coronary artery calcification, the LHC highlighted that it is ‘not just [the] lungs but also [the] heart that is at risk’ [P26/Inc/CS].

#### 3.3.6 Waiting for and undergoing follow‐up tests

Overall, few concerns were raised by those who underwent surveillance for a pulmonary nodule. Knowing that a follow‐up LDCT scan was scheduled or having been psychologically prepared for the possibility of an indeterminate result during the LHC appeared to have provided reassurance. However, for the small minority who did report anxiety, this was significant. One participant described how the ‘result is constantly on my mind’ and how they ‘can't wait for [the follow‐up appointment]… because I can finally put my mind at ease’ [P13/Nod/FS]. The location of the follow‐up appointment being in a cancer clinic caused two participants to infer a cancer diagnosis. Those undergoing diagnostic procedures were understandably more concerned. Of note, one participant reported breathlessness due to anxiety but initially interpreted this as a symptom of lung cancer.

### 3.4 Behavioural responses

The ways in which participants responded behaviourally also varied. Participants predominantly talked about anticipated future behaviours, but a variety of actual changes to behaviour were also mentioned. These are further outlined in this section.

#### 3.4.1 Future anticipated screening participation

The majority intended to take part in any future lung screening programme, with consensus that screening detects lung cancer in its ‘early stages’ [P04/Inc/CS] ‘rather than when it's too late’ [P18/Inc/CS]. Some current smokers described how taking part in regular screening annually or biennially would ‘provide reassurance’ and ‘peace of mind’ [P04/Inc/CS]. However, one participant was uncertain due to their negative experience of their follow‐up test procedure: ‘because of what I've been through, if another letter came… I might have second thoughts’ [P12/Inc/FS].

#### 3.4.2 Future anticipated symptomatic help‐seeking

There was little evidence that taking part in LDCT screening undermined the way participants might respond to future lung cancer symptoms. Some recounted how the LHC had made them more attentive to and ‘conscious about’ [P09/Nod/CS] their lung health. Indeed, many participants, particularly current smokers, indicated that they would promptly seek help from their GP if they were to experience new symptoms, such as ‘a persistent cough’ [P05/Cle/FS]. Furthermore, the LHC appeared to encourage symptomatic help‐seeking, and in one case, raised awareness of previously unacknowledged lung symptoms. For some participants who had not seen their GP for a long time, the LHC and subsequent results provided individuals with an opportunity to see their GP ‘fairly regularly’ and to remain in control of their incidental condition which: ‘stays in the front of my brain’ [P27/Inc/CS]. Nevertheless, a minority described how they anticipated delaying symptomatic presentation, first taking over‐the‐counter medication or only seeking help when they perceived their symptoms as serious. This was because they did not ‘expect the GP to be really expert in lung problems’ [P22/Nod/FS] or because they did not want to be ‘blocking up the system for people that have got something wrong’ [P21/Nod/CS].

#### 3.4.3 Smoking behaviour

Three participants reported that they had stopped smoking either before they attended their LHC (which they set as a date for them to quit: ‘It gave me a focus and an impetus’ [P19/Inc/CS]) or after receiving an indeterminate result. Some participants mentioned how their motivation to quit was increased by the conversation with the nurse at the LHC appointment, an abnormal spirometry reading or the LDCT scan results.

Conversely for some, being diagnosed with an incidental finding, such as chronic obstructive pulmonary disease (COPD) or coronary artery calcification, did not appear to motivate quitting. A few participants cited that this was because they had not been explicitly told to stop and were not concerned enough by the result and because quitting *‘*is all so final’ [P04/Inc/CS]. Others, however, expressed being highly motivated to stop smoking but had low confidence in their ability to quit: ‘I want to give it up, but I really don't think I can’ [P18/Inc/CS]. Instead of quitting, current smokers with an incidental LDCT result tended to report changes to their smoking behaviour, such as cutting down. However, changes in smoking behaviour were not always positive or stable; two participants reported increasing their cigarette consumption while waiting for their LDCT result which appeared to be due to anxiety about their result.

#### 3.4.4 Other health behaviours

Some participants reported engaging more frequently in other cancer prevention behaviours following their LHC and LDCT results including exercise, changes to diet and avoiding air pollution. One participant with an incidental respiratory finding also reported avoiding crowded areas to reduce their risk of contracting respiratory viruses. For some, the LHC resulted in an opportunity to take better care of their health by, for example, going to the GP for regular spirometry tests to help monitor lung function. In some cases, these responses appeared to be perceived as compensatory by those who felt unable to stop smoking but wanted to improve their health: ‘There's this big hurdle I can't get over with the smoking. Everything else I can have a go at’ [P16/Inc/CS].

### 3.5 Factors influencing psychological and behavioural responses

This section outlines the various factors interpreted as influencing participants' responses to the LHC.

#### 3.5.1 Existing concerns about lung health and smoking history

Participants with pre‐existing concerns about symptoms, who acknowledged their smoking status put them at risk of lung cancer or who had lost a family member to cancer, appeared to be more worried about the LHC and their lung health. These concerns appeared to be motivational, with symptoms such as breathlessness being described as the reason for attending the LHC: *‘*I was over 60… I did want a lung function test because I was having difficulty breathing’ [P02/Inc/FS]. Existing concerns also appeared to foster positive emotional responses to the subsequent LDCT result; these participants frequently described ‘relief’, as well as strong intentions to seek help promptly for future symptoms.

On the other hand, a lack of pre‐existing concern or symptoms was linked to a corresponding lack of concern about the upcoming LHC ‘I didn't really feel concerned… I haven't seen any symptoms or anything of any kind of chest problems’ [P08/Nod/FS], feeling ‘sanguine’ while waiting for the LDCT result [P10/Nod/FS] and expecting the LDCT result to ‘show nothing’ [P08/Nod/FS]. Participants who perceived themselves as asymptomatic (not having ‘any obvious lung problems, shortness of breath or anything like that’ [P22/Nod/FS]) or as having a less significant smoking history, (eg ‘never used to smoke so hard’ [P06/Nod/CS], described thinking about their lung health only ‘fleetingly, on very rare occasions’ [P25/Nod/CS], if at all. A lack of concern due to potential misattribution of symptoms to ‘the quality of the cigarettes nowadays’ also appeared to adversely affect future help‐seeking intentions.

Interestingly, prior concern may have been instrumental in whether an abnormal LDCT result motivated behaviour change. Pre‐existing concern about health appeared to motivate positive behaviour change regardless of the type of LDCT result, including motivation to quit or reporting (temporarily) cutting down smoking. However, for those with no prior concerns, an indeterminate result alone did not appear to be sufficient to motivate change in smoking behaviour with two participants noting how they had started to smoke more after temporarily cutting down and appeared to be more likely to engage in compensatory behaviours.

#### 3.5.2 Social support

Participants with partners frequently discussed their LHC invitation with them who often encouraged attendance: ‘we both agreed that it would be a good idea’ [P13/Nod/FS]. Similarly, one initially reluctant participant had shown the invitation to a community group worker who suggested they attend: ‘I was hesitant and […] she said, surely, it's better to know’ [P28/Inc/CS]. For some, social support appeared to be influential throughout the screening pathway, including buffering well‐being through emotional support, aiding comprehension of LDCT results and supporting positive behaviour change. However, some participants did not discuss their LHC with anyone, explaining that they ‘don't discuss health problems’ [P02/Inc/FS] or how they ‘didn't want to worry anyone’ [P23/Nod/CS].

#### 3.5.3 Stigma and self‐blame

Links between stigma and smoking were evident: ‘they [healthcare professionals] are not going to bother with people who smoke’ [P20/Nod/CS]. Notably, two participants displayed feelings of guilt to the extent that they felt they ‘deserved’ lung cancer. These perceptions appeared to provoke greater worry about the potential LDCT result, higher affective risk perceptions and lack of reassurance from a LDCT result showing no lung cancer. Indeed, some participants expressed gratitude for receiving an incidental finding: ‘it makes me feel that I was quite lucky’ [P12/Inc/FS], while another remained pessimistic, thinking that a lung cancer diagnosis was only ‘a matter of time’ [P16/Inc/CS].

#### 3.5.4 Negativity and fatalism

Current smokers, in particular, held negative views about the state of their respiratory health which seemed to foster worry about LDCT results and higher perceived risk of lung cancer. Concern about irreversible harm caused by smoking meant many ‘expected the worst’ [P15/Nod/FS], with one participant fatalistically accepting that ‘there is lung cancer in my future somewhere’ [P04/Inc/CS]. Lung cancer was perceived as a death sentence by some participants, with one explaining that the word ‘cancer means death’ to their generation [P16/Inc/CS]. There was evidence that this negativity led to hesitancy in seeking social support for a follow‐up appointment, because ‘cancer is such an awful thing’ [P23/Nod/CS].

However, an initially negative and fatalistic outlook appeared to lead to positive psychological responses following the LDCT result, including feeling ‘pleased’ [P21/Nod/CS] or ‘relieved’ [P04, Incidental, CS]. Furthermore, four of these participants changed their smoking behaviour, either by temporarily cutting down or by attempting to quit; suggesting that the contrast of positive news against fatalistic expectations could be a catalyst for motivating positive health behaviour change.

#### 3.5.5 Competing Priorities

Two participants were focused on their other existing medical conditions and consequently the LDCT results were considered relatively unimportant: ‘I've got so many things wrong with me… just another thing’ [P12/Inc/FS], whereas another individual diagnosed with an indeterminate result revealed that they thought ‘about it more because I wasn't expecting it to be a cause for concern’ [P10/Nod/FS]. Similarly, others mentioned that the invitation to the LHC, although it was readily embraced and acted upon, came when they had been faced with challenging life circumstances such as a daughter's cancer diagnosis.

## 4 DISCUSSION

This study broadens our understanding of the psychological and behavioural impact of LDCT lung cancer screening. We identified a more diverse spectrum of responses experienced throughout all stages of the screening pathway than has been described previously. As expected, these varied widely encompassing positive responses as well as negative responses, with wider‐reaching anticipated implications for future prevention and early detection behaviour. Individual differences in response appeared to be influenced by the type of LDCT result, existing concerns and expectations about health, negative beliefs and perceived stigma, as well as social support and competing priorities.

We observed psychological responses to all stages of the screening pathway, not just the LDCT results, including the invitation letter, communication of risk‐based LDCT eligibility and the spirometry test. Importantly, these responses appeared to have implications for how individuals subsequently responded to their LDCT screening test and results. For example, the type of spirometry result received (ie normal or abnormal) generated an emotional response which seemed to either positively or negatively affect individuals' perceptions of their lung cancer risk, their motivation to stop smoking, their expectations for their LDCT result and their degree of concern about cancer after receiving an indeterminate LDCT result. Similarly, the initial invitation letter appeared to instigate varied responses including quit attempts, heightened attention to respiratory symptoms, empowerment over one's respiratory health and raised concern among those with fatalistic perceptions of lung cancer. While we could have organized the psychological responses in a different way, presenting these findings by stage highlights the importance of evidence‐based communication at earlier stages of the screening pathway too (eg at first invitation and during the preceding LHC), when there may be potential to psychologically prepare individuals for the different LDCT results as well as opportunities to promote behaviour change. Further research is needed to understand the ways in which these responses interact across different stages of the screening pathway.

Regarding the impact of LDCT results more specifically, existing studies have found that receiving an ‘all‐clear’ result after investigations for a suspicious symptom or a negative bowel cancer screening result increases the likelihood of individuals appraising subsequent symptoms as benign^16^, ^17^ and delaying help‐seeking. In the present study, participants reported becoming more attentive to symptoms and their respiratory health, with only a minority expressing lower concern about future symptoms. Current smokers appeared to be most likely to anticipate seeking help promptly; a group that has been found to be less likely to seek help for lung cancer symptoms compared with non‐smokers in previous research.^21^ Our participants' accounts were hypothetical, and those who attend screening may be more proactive in seeking help than the general population. Nevertheless, these findings suggest there may be something about the screening process that fosters symptom awareness, which could provide an opportunity to encourage prompt presentation among a high‐risk group.

Participants with indeterminate results reported increases in distress consistent with previous research.^6^, ^8^, ^22^ However, there was an apparent lack of concern about incidental findings (eg COPD) among some participants who were understandably pleased their scan showed no sign of lung cancer but in some cases regarded this an ‘all‐clear’ for their respiratory health. This suggests that incidental findings carry low risk of psychological distress which is reassuring, but also implies risk of over‐reassurance akin to ‘clear’ screening results raised by a previous study.^23^ Indeed, current smokers with an incidental finding more frequently reported cutting down on smoking rather than making a quit attempt compared with those who had indeterminate results. Communicating incidental results may therefore be an opportunity to capitalize on the initially positive emotional response to motivate positive behaviour change that could ultimately halt disease progression.

Indeed, our findings suggest the LDCT screening pathway could provide multiple opportunities to support cancer prevention. Those participants, who stopped smoking, did so at different points along the pathway including after receiving the initial invitation letter as well as following indeterminate results. There was also evidence of improvements in exercise and diet supporting findings from a previous US qualitative study.^13^ Indeed, other studies have proposed cancer screening as a largely acceptable context for providing advice about multiple behavioural risk factors^24^ and some degree of behaviour change was observed without intervention by one study.^25^ Integrating a broader cancer prevention approach may be especially beneficial for lung cancer screening participants given existing data showing that behavioural risk factors cluster. For example, those who smoke are also less likely to be physically active or drink within recommended alcohol limits.^26^, ^27^ Further research is needed to quantify these responses and understand how to best provide and integrate advice and signposting to support within lung screening services.

While this study focused on the psychological and behavioural responses that high‐risk individuals had to screening, there are other possible types of consequences that were not studied. These could include physical (eg exposure to radiation) or financial harms, as proposed in Harris and colleagues' taxonomy.^28^ Future work will be needed to explore how these other types of harm might affect individuals' psychological and behavioural responses along the screening pathway. We used purposive sampling and recruited from a ‘real‐world’ demonstration pilot of lung cancer screening; strategies intended to increase the ecological validity of our results. Nevertheless, it should be acknowledged that LSUT participants may differ from the wider lung screening population as the eligibility criteria were purposefully narrower; recruiting a relatively older (aged 60‐75) cohort of predominantly current smokers and recently quit former smokers living within the two most deprived quintiles nationally. This means the data reported here may not fully reflect the range of views and experiences of lung screening attenders. Our sample is also likely to be subject to self‐selection bias as our participants volunteered to be interviewed. We cannot rule out the possibility that those who did not take part would have reported different responses to lung screening. Furthermore, while a minority of our participants received a clear LDCT result, we focused on individuals with indeterminate and incidental findings, and so further research is needed to better understand responses among those who receive a clear screening result. Finally, our findings may be subject to recall bias, which may have led participants to recall fewer responses and of a lower intensity.

In conclusion, the ways in which individuals respond to LDCT screening both psychologically and behaviourally are more diverse than have been described by previous studies and span the entire pathway, beginning with the screening invitation. The ways in which screening is delivered by health‐care professionals and communicated to participants should therefore be evidence‐based and patient‐centred at every stage, not just at the LDCT test. Importantly, negative responses may be reduced through psychological preparation for the different types of screening results and there is potential to capitalize on positive responses to support positive behaviour change in cancer prevention, symptom awareness and screening adherence. The present findings should help direct a broader psychological research agenda driven to optimize patient benefit from LDCT lung cancer screening.

## CONFLICT OF INTEREST

SK, JW, JC and SLQ declare no conflicts of interest. MR and SMJ are supported by funding from a commercial US health‐care company (GRAIL Inc) as part of funding for a large trial of low‐dose CT screening, called the ‘SUMMIT Study’. SQ collaborates on the SUMMIT Study. SMJ has been paid by Astra Zeneca, BARD1 Bioscience and Achilles Therapeutics for being an Advisory Board Expert and travel to one US conference. SMJ receives grant funding from Owlstone for a separate research study. MR has received travel funding for a conference from Takeda and an honorarium for speaking at an educational meeting from Astra Zenica. All authors perceive that these disclosures pose no academic conflict for this study. All authors declare no other relationships or activities that could appear to have influenced the submitted work.

## Data Availability

The data that support the findings of this study are available from the corresponding author upon reasonable request.
