# Supplementary material for: Mapping the spectrum of psychological and behavioural responses to low‐dose CT lung cancer screening offered within a Lung Health Check
Source: Health Expect. 2020 Jan 21;23(2):433–41. doi: 10.1111/hex.13030 (PMC7104654; doi:10.1111/hex.13030)
Supplement: Supplementary file 1 [file HEX-23-433-s001.doc]

**Supplementary File 1:** Coding frame

| **CHART 1: BACKGROUND**  Study ID number  1 Age  2 Gender  3 Smoking status  4 Socioeconomic status  **CHART 2: PSYCHOLOGICAL RESPONSES BEFORE**  **LDCT SCAN**  Identifier  1 Feelings and thoughts in response to invitation letter  2 Lung Health Check as means of being cared for  3 Perceptions of lung health and lung cancer risk  4 Feelings and thoughts about screening eligibility  5 Feelings and thoughts about spirometry test  5 Thoughts about smoking  6 Misc/other  **CHART 3: PSYCHOLOGICAL RESPONSES AFTER**  **LDCT SCAN**  Identifier  1 Feelings and thoughts when waiting for LDCT results  2 Feelings and thoughts when receiving LDCT results  3 Perceptions of lung health and lung cancer risk  4 Feelings and thoughts about having Lung Health Check  5 Feelings and thoughts about having LDCT scan  6 Expectation of LDCT scan result  7 Feelings and thoughts when waiting for follow-up tests  8 Perceptions of control over health  9 Significance of incidental finding relative to cancer  8 Misc/other  **CHART 4: BEHAVIOURAL RESPONSES**  Identifier  1 Intention to take part in future lung screening programme  2 Motivation to quit smoking  3 Changes/no changes in smoking behaviour  4 Attempts to stop smoking  5 Changes in other health behaviour/lifestyle  6 Perception of future  7 Focus on health and other areas of health  9 Other/misc. | **CHART 5: POTENTIAL INFLUENCES ON PSYCHOLOGICAL AND BEHAVIOURAL RESPONSES**  Identifier  1 Previous/current concerns about health or symptoms  2 Social networks and support  3 Negative perceptions (i.e. stigma, fatalism)  4 Comorbidities and other health problems  5 Other life commitments  6 Dispositional approach and style of coping  7 Perceptions of smoking  8 Optimistic bias about health  9 Misc/other |
| --- | --- |
